# Preferences for nutrition education delivery among low-income older adults

**DOI:** 10.1080/07853890.2026.2722716

**Published:** 2026-08-25

**Authors:** Khary K. Rigg, Steven L. Proctor, Kayleigh B. Faber, Tara Tyler

**Affiliations:** ^a^ Department of Behavioral Health Science & Practice, University of South Florida, Tampa, FL, USA; ^b^ Thriving Mind South Florida, Miami, FL, USA; ^c^ Department of Psychiatry and Behavioral Health, Herbert Wertheim College of Medicine, Florida International University, Miami, FL, USA

**Keywords:** Nutrition education, low income, older adults, intervention delivery preferences

## Abstract

**Objective:**

Older adults with limited financial resources often experience barriers to accessing effective nutrition education, which can contribute to poorer dietary and health outcomes. This study aimed to identify preferred approaches for delivering nutrition education among low-income older adults.

**Methods:**

Online surveys and in-depth qualitative interviews were conducted with 15 adults aged 65 years and older with annual incomes below $40,000. Surveys collected sociodemographic information and were administered using Qualtrics, with descriptive analyses conducted in IBM SPSS Statistics. Interviews explored preferences for nutrition education delivery and were analyzed thematically using NVivo to identify recurring patterns and themes.

**Results:**

Participants strongly preferred in-person nutrition education delivered in familiar, community-based settings. Preferences were driven by accessibility, established routines, and opportunities for relationship building. Social interaction with peers was viewed as an essential component of effective learning and engagement. Hands-on instructional approaches, including food demonstrations and taste tests, were perceived as especially engaging and motivating. In contrast, virtual formats were generally viewed less favorably due to digital literacy challenges, limited technology access, and diminished personal connection.

**Discussion:**

This study provides insight into how nutrition education can be more effectively delivered to low-income older adults, particularly those facing economic challenges. Findings suggest that in-person, community-based programming emphasizing social interaction and experiential learning may improve engagement and perceived effectiveness. At the same time, barriers associated with virtual delivery formats highlight important limitations of increasingly technology-driven nutrition education approaches for this population.

## Introduction

Adults aged 65 years and older represent one of the fastest-growing segments of the United States population, a demographic shift that has heightened the need for effective interventions to support healthy aging [1]. Nutrition plays a central role in maintaining health and wellbeing during later life [2]. Healthy eating habits contribute to the prevention and management of several chronic health conditions such as cardiovascular disease, diabetes, and obesity, all of which are prevalent among older adults [3]. Despite the importance of proper nutrition, many older adults face significant barriers to maintaining healthy diets, including limited income and transportation [4].

These challenges are particularly pronounced among older adults with limited financial resources. Low-income older adults are more likely to experience food insecurity and low nutrition literacy, which are associated with poorer health outcomes [5]. Financial limitations may contribute to greater reliance on lower-cost food options, which are often less nutrient-dense than healthier alternatives. Together, these factors may place economically disadvantaged older adults at increased risk for poorer diet quality and adverse health outcomes [6]. Targeted nutrition education programs may, therefore, be particularly important for this population.

Nutrition education programs have long been recognized as an effective strategy for promoting healthier dietary behaviors [7]. Such programs typically aim to improve knowledge about healthy eating, increase awareness of nutrition guidelines, and develop practical skills related to meal preparation and food selection [1]. Many community-based interventions have demonstrated that nutrition education can lead to meaningful improvements in dietary behaviors among older adults [8]. However, the success of nutrition education interventions depends not only on the quality of the information provided, but also on the manner in which programs are delivered [9]. Program accessibility and participant engagement are strongly influenced by delivery methods, including whether programs are conducted in person, online, or through other formats [10]. Understanding how participants prefer to receive nutrition education is, therefore, critical for designing effective interventions.

Historically, nutrition education programs for older adults have been delivered in community-based settings such as senior centers, community health clinics, and faith-based organizations [11]. These settings offer opportunities for social interaction, peer learning, and hands-on activities, such as cooking demonstrations. In recent years, however, the expansion of digital technologies has created new opportunities for delivering nutrition education through online platforms [12]. Virtual programming can increase the reach of interventions and reduce logistical barriers related to transportation or scheduling [13,14]. While technology-mediated approaches offer certain advantages, they may not be equally accessible to all populations, and the extent to which older adults are receptive to these approaches is not well understood.

Many older adults experience challenges accessing and using digital technologies, such as computers, smartphones, and online platforms, and research suggests these barriers are especially common among those with fewer financial resources [15]. These challenges contribute to what is commonly described as the ‘digital divide’, which disproportionately affects older adults and individuals with lower socioeconomic status [16]. Technology-based approaches to nutrition education may therefore be less accessible for some older adults, particularly those with limited financial resources or digital literacy skills. Delivery method preferences among older adults remain understudied, however, and relatively little is known about how they prefer to receive nutrition education, whether through in-person, virtual, or other formats. Many nutrition education interventions targeting this population continue to be informed by evidence derived from younger populations [17]. Programs developed without direct input from older adults may not fully reflect their unique needs and preferences. A better understanding of how low-income older adults prefer nutrition education to be delivered may help inform the development of interventions that are more accessible and responsive to the challenges they face. Programs that align with participant preferences may be more likely to promote participation and sustained engagement.

## Current study

Previous research has examined nutrition education interventions for older adults, including community-based programs and digital delivery approaches [8,12,14]. However, less is known about how low-income older adults prefer nutrition education to be delivered. Understanding these preferences is important because the mode of delivery can influence both program accessibility and participant engagement. This study explored nutrition education delivery preferences among low-income older adults in Florida, a state with one of the nation’s largest and fastest-growing older adult populations. This subgroup was selected because economically disadvantaged older adults are particularly vulnerable to food insecurity, financial constraints, and other barriers to healthy eating [18]. Specifically, the study aimed to identify preferred program formats and characteristics that participants believed would enhance accessibility and engagement. A qualitative approach was used because these preferences are often nuanced, context-dependent, and best understood through in-depth exploration.

## Methods

### Participants

Study data were drawn from a larger program evaluation of a nutrition education intervention funded by a grant from the Humana Foundation. As part of this evaluation, participants completed qualitative interviews before participating in the nutrition education intervention. This subset of data was used for the present study. Therefore, participants’ responses reflected their preferences and expectations regarding nutrition education delivery rather than experiences with the intervention itself. Data were collected from 15 participants in the Tampa Bay region of Florida, a largely metropolitan area. To be eligible, participants had to be at least 65 years of age, have a behavioral health diagnosis, and an annual income below $40,000. The activities described in this study were reviewed by the University of South Florida Institutional Review Board (Study #007789) and were determined to be program evaluation, and therefore did not meet the definition of human subjects research. The study was conducted in line with the principles of the Declaration of Helsinki.

### Procedure

Purposive sampling strategies were used to recruit participants. Recruitment occurred between March 2025 and April 2025 through the distribution of flyers and study information cards at senior centers and residential facilities serving older adults. These settings were selected because they regularly serve older adults who were likely to meet the study eligibility criteria and represent common locations where nutrition education programs are delivered. Chain referral methods were also used, whereby participants who completed the study could refer other potentially eligible individuals. However, no incentives were provided for referrals. Interested individuals were instructed to contact a member of the study team and complete a brief eligibility screening conducted by phone. During the screening, participants were asked to verify eligibility-related information, including their age and annual income, to determine whether they met the study inclusion criteria.

Once eligibility was confirmed, participants were provided with an online consent form to review and sign. After consent was obtained, a study team member scheduled a 60-minute interview conducted *via* Microsoft Teams. Participants were given the option of joining the interview either through a Microsoft Teams meeting link sent to their email address or by calling into the meeting using a telephone number. Offering both video- and telephone-based participation options helped accommodate older adults with varying levels of comfort with videoconferencing platforms. Individual virtual interviews were selected because they allowed for in-depth exploration of participants’ nutrition education delivery preferences while reducing participant burden. All interviews were conducted by the first author. During the first 10 min of the interview, participants completed a brief survey assessing demographic (e.g. age, race, income, education, gender) and psychosocial characteristics (e.g. behavioral health diagnoses, housing status, insurance coverage), with responses entered directly into Qualtrics by the interviewer. The remainder of the session consisted of a semi-structured qualitative interview exploring participants’ preferences for nutrition education delivery. The interview guide was developed and reviewed by a Community Advisory Board, consisting of a low-income older adult, a registered dietitian, and professionals serving older adults. Interviews began with broad, open-ended questions to build rapport and orient participants to the discussion. Participants were then asked more focused questions regarding nutrition education delivery preferences before concluding with an opportunity to share any additional comments. Participants were asked open-ended questions about preferred program formats and characteristics that would make programs more accessible (e.g. ‘How would you prefer to receive information about healthy eating?’ ‘What might make it difficult to participate in a nutrition education program?’ ‘What would make a program useful and easy to attend?’). Participants received a $100 Walmart e-gift card for their participation. To protect participant confidentiality, identifying information was removed from study materials, and all electronic data, including audio recordings and transcripts, were stored on secure, password-protected university servers accessible only to authorized study personnel.

### Analyses

For the demographic survey data, descriptive statistics were calculated, with percentages reported for categorical variables and means for continuous variables. All quantitative analyses were conducted using SPSS statistical software [19]. For the qualitative portion, each interview was audio-recorded and transcribed using Microsoft Teams. The first author reviewed each transcript against the original recording and corrected any transcription errors before analysis. The transcripts were then imported into NVivo (Version 14) for analysis [20].

The data were analyzed using inductive thematic analysis, an approach well suited for identifying and describing patterns across participants’ experiences and perspectives [21]. The first author, who has extensive training and experience in qualitative research methods, conducted all stages of the analysis, including coding, theme development, and interpretation. Using a single analyst promoted consistency in the application of codes and facilitated deep familiarity with the data. To enhance trustworthiness, the first author maintained analytic memos throughout the coding process and reviewed transcripts multiple times. Emerging codes, themes, and interpretations were also discussed with other members of the research team to help ensure that findings were grounded in the data and to challenge potential biases.

The following paragraph provides a brief overview of the six-phase process used to conduct the thematic analysis [21]. Phase one involved becoming familiar with the data by reading each transcript twice, with initial ideas for coding noted during the second reading using the software’s memo feature. In phase two, initial codes were generated, and delivery-preference content was systematically coded across the entire data set. Once coding was complete, phase three focused on organizing these codes into potential themes and collating all relevant excerpts for each theme. Phase four involved reviewing and refining the themes to ensure that the data within each theme cohered together into a meaningful pattern. In phase five, themes were clearly defined and named, and the overall narrative of the findings was developed. In the sixth and final phase, the report was written, and illustrative participant excerpts were selected to support each theme. Saturation was reached after 15 interviews, as no new patterns emerged in the data. Themes are presented in a logical sequence to facilitate interpretation and should not be viewed as reflecting their relative importance.

## Results

### Sample characteristics

Demographic characteristics of the sample are shown in Table 1. Participants ranged in age from 65 to 81 years, with an average age of about 70. The sample was predominantly female (87%) and mostly White (53%). Most were fully retired (73%), though a few were working part-time or seeking employment. Two-thirds reported annual incomes of $19,999 or less. Most lived in condominiums or apartments (73%), while the remainder lived in single-family homes or mobile homes. Two-thirds had a high school diploma/GED, while the rest held bachelor’s or graduate degrees. Only 13% were married, while the remaining participants reported being nonmarried (i.e. divorced, separated, widowed, or never married). The following section describes the qualitative themes that emerged regarding delivery preferences.

**Table 1.** Demographic and socioeconomic characteristics of interview participants (*n* = 15).

Measure	n (%)
**Age (M)**	
Average age of all participants (65–81)	69.7
**Gender**	
Female	13 (87)
Male	2 (13)
**Race**	
White	8 (53)
Black	6 (40)
Asian/Pacific Islander/Native Hawaiian	1 (7)
**Employment Status**	
Fully retired	11 (73)
Working part-time	3 (20)
Unemployed, looking for work	1 (7)
**Annual Household Income**	
$0–$9,999	2 (13)
$10,000–$19,999	7 (47)
$20,000–$29,999	3 (20)
$30,000–$39,999	3 (20)
**Housing**	
Single-family detached home	2 (13)
Condominium or apartment (units above or below)	11 (73)
Mobile home	2 (13)
**Education**	
High school diploma or GED	10 (67)
Bachelor’s degree	4 (26)
Graduate degree (e.g. master’s, doctoral degree)	1 (7)
**Marital Status**	
Married	2 (13)
Divorced, separated, or widowed	11 (73)
Never married	2 (13)

*Note.* M = mean. Percentages may not sum to 100 due to rounding.

### Qualitative findings

#### Community-based in-person preferences

Participants consistently expressed a strong preference for nutrition education delivered in person within familiar, trusted community-based settings, such as senior centers or churches. In-person programming was perceived as more accessible and engaging. Many participants emphasized that being physically present in a shared space made learning feel more tangible and supported sustained attention compared to phone- or video-based formats. Familiar community-based locations were viewed as places where participants already felt comfortable and safe, which increased perceived feasibility and willingness to attend sessions regularly. This participant described some of the reasons why he preferred in-person environments to virtual formats:

‘I think face-to-face is way better than virtual … because being virtual is gonna make people not pay attention. It’s gonna make them miss a lot of stuff … but if you have somebody there that’s face-to-face, hands on, it is much better, especially for seniors … It’s much better to just be hands on. You’re right there and I can see the facilitator, and they can see me. I can also ask questions to understand things better if I don’t understand what is being taught’. (68-year-old male)

Beyond general preference for face-to-face delivery, participants further described community-based settings as uniquely supportive of both learning and attendance. Familiar locations reduced uncertainty related to transportation and social comfort, particularly for individuals with mobility limitations or health conditions. Participants noted that attending sessions in familiar spaces, such as senior centers with existing activities, made participation feel more routine and worthwhile. This participant describes the appeal of delivering programs in comfortable, community-based settings:

‘If it’s delivered somewhere I already know, I don’t really worry about it. I know how to get there and I know what it’s gonna be like. That makes it easier for me to come, especially when you’re dealing with buses and all that … If it’s somewhere you’re comfortable, you’re more likely to go. You’re not worried about how the place is. Familiar places just make things easier’. (65-year-old female)

#### Social interaction matters

Participants described social interaction with peers as a meaningful component of effective programming. Many emphasized the importance of being around other older adults, sharing experiences, exchanging perspectives, and learning alongside individuals facing similar circumstances. These interactions were viewed as sources of emotional support that reinforced participation beyond the educational content alone. Group-based formats were perceived as fostering a sense of connection and companionship that enhanced the overall experience and made participation feel purposeful. As one participant described, the value of peer interaction lies in the exposure to the shared experiences of other older adults:

‘I like being in a group because you hear how other people do things. Sometimes somebody says something and you think, ‘I never thought about it that way,’ and that helps … Socializing with other seniors is important. We all got things going on, and just talking and listening to each other helps you learn’. (70-year-old female)

In addition to peer engagement, participants highlighted the importance of direct interaction with facilitators and the value of leaving their homes to attend programs in person. Being physically present in shared spaces was seen as an opportunity to personally engage with the facilitator, rather than passively receive information from them. Additionally, participants noted that attending group sessions helped break routines of isolation and provide social stimulation. For individuals living alone or with limited daily interaction, simply getting out of the house was perceived as beneficial for emotional well-being and motivation to attend consistently. As one participant stated:

‘Being around other seniors helps, because you can hear what they’re going through too. You realize you’re not the only one dealing with stuff like this, and that makes you feel better … People like being together. When you’re in a group, you get different opinions and can actually see the facilitator talking, and that makes it more interesting. It makes people want to come back’. (73-year-old female)

#### Hands-on learning preferred

Participants consistently expressed enthusiasm for hands-on learning components, describing interactive activities such as food demonstrations, label reading exercises, and taste tests as especially engaging. These experiential elements were viewed as more effective than lecture-based instruction because they allowed participants to actively participate and apply information immediately. Hands-on activities were perceived as holding attention longer and making the content more memorable. Participants emphasized that learning by doing (not just listening) made nutrition information easier to understand and use in daily life. This participant explains as follows:

‘I like when you can actually see it and try it. Like when you bring the food in and we can taste it or look at it together, that helps me understand it better. When you can put your hands on it, it sticks with you more’. (65-year-old female)

Participants also described hands-on activities as confidence-building and motivating, particularly when learning about unfamiliar foods or preparation methods. Taste tests and demonstrations were viewed as low-pressure opportunities to explore healthier options and reduce hesitation around dietary changes being discussed. Several participants noted that trying foods during sessions increased their willingness to replicate those behaviors at home, especially when guided by a facilitator and supported by peers. These experiential components were also perceived as being ‘fun’, which participants felt would help reinforce attendance and sustain interest in the program. As one participant described, hands-on approaches make learning not only approachable, but actionable:

‘When you bring the snacks and we all try them, it makes a difference. Then I know, ‘okay, I can eat this,’ and it’s not just somebody telling me about it. Tasting it and seeing how it’s done makes me feel like I can actually do this at home’. (73-year-old female)

#### Barriers to virtual formats

Participants overwhelmingly identified video-based and virtual formats as challenging, citing limited access to technology and low digital literacy as major barriers. Several participants reported not owning computers, lacking reliable internet access, or feeling uncomfortable using video-based platforms altogether. Even among those who owned smartphones or tablets, many described minimal experience with video calls and expressed uncertainty about navigating virtual platforms without assistance. Participants emphasized that these technological barriers would be likely to deter their participation entirely. As one participant explained:

‘I don’t have a computer, first of all, because I can’t afford it. I got a little tablet, but not a real computer, and I don’t do that much computer stuff anyway. I’m just not into that, so I stay away from it … I don’t even use video chat on my phone. I’m just not into all that computer stuff’. (81-year-old female)

In addition to technical challenges, participants identified several practical concerns that made virtual formats less appealing. Many described difficulties sustaining attention during virtual sessions, noting that distractions at home made disengagement more likely. Others expressed concerns about managing technical issues independently, including navigating links and troubleshooting audio-visual problems during sessions. Participants also questioned how virtual programs could adequately support experiential components, such as taste tests or food demonstrations. Collectively, these concerns contributed to skepticism about whether virtual formats were an effective approach for older adults. One participant summarized these concerns as follows:

‘Virtual is gonna make it hard for people to pay attention. They’re gonna be getting up, doing other stuff, or just tuning you out, because when you’re at home there’s too many distractions … and if you start having problems with the computer or the sound or whatever, a lot of people aren’t gonna know how to fix that … and taste testing or actually seeing the food, you can’t really do that the same way online’. (68-year-old male)

## Discussion

This study helps address a critical gap in the literature by examining how older adults, particularly those who are low-income, prefer to engage with nutrition education. While existing research has largely emphasized their nutritional needs, far less attention has been paid to the delivery preferences of high-risk subpopulations of older adults [22]. By centering the perspectives of individuals experiencing both age- and income-related vulnerabilities, this study provides novel insight into how nutrition education can be delivered more effectively.

A key finding of this study is the strong preference for community-based, in-person nutrition education delivered in familiar and trusted settings. This finding aligns with prior research suggesting that community-based environments can enhance reach and engagement among older adults by providing accessible, trusted spaces for health promotion activities [23,24]. Participants also emphasized the importance of familiar locations, a finding consistent with previous research showing that transportation challenges and uncertainty about unfamiliar environments can hinder participation among older adults [25,26]. These findings further suggest that referrals to nutrition education programs may be more effective when providers connect individuals to services embedded within settings they already know and trust.

Participants consistently described familiar community-based settings as more accessible and appealing than other delivery options. This finding is consistent with prior research highlighting the value of community-based programs for engaging older adults in health promotion activities [27,28]. When programs are embedded within settings that older adults already frequent, participation may feel more convenient and less burdensome [29]. These findings suggest that place-based approaches may help reduce barriers to engagement and increase the likelihood of participation. Policymakers should consider prioritizing funding mechanisms that support community-based interventions and the co-location of services, particularly in underserved communities where older adults may face multiple barriers to care.

Another central finding is the importance of social interaction as a core component of effective nutrition education. Participants consistently emphasized the value of learning alongside peers, sharing experiences, and engaging with the facilitator in person. This finding is consistent with prior research highlighting the benefits of social connectedness and group-based programming for older adults [30–34]. The social nature of these interactions may help participants contextualize nutrition information through the exchange of lived experiences, making it feel more relevant and actionable and potentially increasing the likelihood of behavior change [35]. At a programmatic level, these findings suggest that nutrition education interventions should be intentionally designed to facilitate dialogue, peer support, and shared learning, rather than relying solely on didactic instruction. This recommendation is consistent with prior guidance emphasizing the importance of interactive, participant-centered approaches in nutrition education programs for older adults [36].

Participants’ strong preference for hands-on learning further reinforces the importance of active engagement in nutrition education. This finding is consistent with prior research suggesting that experiential and hands-on approaches can enhance participant engagement and learning in nutrition education programs [37]. Activities such as food demonstrations, taste tests, and label-reading exercises were perceived as more effective than passive, lecture-based approaches. This suggests that experiential learning may enhance retention and self-efficacy by allowing individuals to immediately apply new knowledge [38,39]. This approach may be particularly beneficial for older adults, who often benefit from concrete, practical demonstrations that can help translate abstract dietary recommendations into actionable behaviors [40]. Findings also indicate that hands-on activities may play a critical role in reducing uncertainty and building confidence around dietary change. Participants described the opportunity to taste unfamiliar foods and directly observe preparation techniques as helping to demystify healthier options and increasing their willingness to adopt them in their daily lives.

In contrast, participants expressed strong aversion to virtual formats. These findings are consistent with prior research documenting persistent barriers related to technology access and digital literacy among economically disadvantaged older adults [15,41]. Participants’ descriptions suggest technology-related barriers may limit participation in virtual nutrition interventions for some older adults. This finding aligns with previous studies reporting that limited digital access and technology literacy can reduce older adults’ participation in telehealth and other digitally delivered health interventions [42,43]. Although telehealth and virtual care have expanded considerably in recent years, prior research suggests that hybrid models combining in-person and virtual components, along with digital literacy support, may improve accessibility for older adults [44,45]. However, consistent with our findings, other studies have found that many older adults continue to prefer in-person programs because they foster greater engagement, accountability, and social connection [46]. Taken together, these findings suggest that in-person delivery should remain a central component of nutrition education programming for low-income older adults, while hybrid approaches may offer additional flexibility for those who are comfortable using technology.

Many of the preferences identified in this study are consistent with existing evidence and practice recommendations for nutrition education programs targeting older adults [30,36]. Prior research has highlighted the benefits of group-based programming, social interaction, and active participation, while professional guidelines recommend incorporating these elements into community nutrition programs [40, 42, 47]. The contribution of the present study, however, extends beyond these recommendations. By capturing the perspectives of low-income older adults directly, the findings help explain why these program characteristics are valued and identify implementation considerations that may influence participation and engagement. Participants emphasized the importance of familiar community settings, perceived accessibility, and opportunities for social connection, while also highlighting barriers associated with virtual delivery. These insights provide important context for translating existing recommendations into practice and may help guide the design of more accessible and responsive nutrition education programs for low-income older adults.

Several limitations should be considered when interpreting these findings. The sample was relatively small and predominantly female, and findings should be interpreted with this in mind. Because individual interviews were used to collect data, social desirability and interviewer bias may have occurred. However, these effects are believed to have been mitigated through the use of an experienced interviewer. An additional limitation is that participation required completion of an online consent form and Microsoft Teams interview. Although participants were offered the option of joining interviews either through a Teams meeting link or by telephone, participation still required some degree of technology access and familiarity. As a result, individuals experiencing the most substantial technology-related barriers may have been less likely to participate. Future research should test whether the preferences described in this study actually result in increased efficacy and/or behavior changes. Research should also examine ways to reduce barriers to virtual formats, including hybrid models and digital literacy support.

## Conclusion

In summary, this study provides important insight into how nutrition education can be more effectively delivered for older adults, particularly those facing economic challenges. Findings point to a clear preference for in-person, community-based programming delivered in familiar settings, with strong emphasis on social interaction and hands-on learning. At the same time, significant barriers to virtual formats highlight the limitations of technology-driven approaches for this population. Aligning interventions with these preferences has the potential to improve program effectiveness and ultimately support healthy aging among older adults.

## Data Availability

Due to the qualitative nature of this study and the potential risk of participant identification, the data are not publicly available.
